# The Role of Chlamydia pneumoniae in the Aetiology of Autoimmune Diseases

**DOI:** 10.7759/cureus.49095

**Published:** 2023-11-20

**Authors:** Basant K Puri, Gary S Lee, Armin Schwarzbach

**Affiliations:** 1 Health and Wellbeing, University of Winchester, Winchester, GBR; 2 Molecular Biology, Cambridge Advanced Research, Cambridge, GBR; 3 Medicine, University of Southampton, Southampton, GBR; 4 Pathology, ArminLabs, Augsburg, DEU

**Keywords:** immunoglobulin a, heat shock protein 60, autoimmunity, anticyclic citrullinated peptide antibodies, chlamydia pneumoniae

## Abstract

Introduction

The most prevalent chronic human autoimmune disorder worldwide is rheumatoid arthritis. Synovial samples from acute-phase patients are polymerase chain reaction-positive for *Chlamydia pneumoniae *(*C. pneumoniae*)* *DNA and express chlamydial *hsp60*. Furthermore, anti-cyclic citrullinated peptide (anti-CCP) antibodies promote apoptosis of mature human Saos-2 osteoblasts via cell surface binding to citrullinated heat shock protein 60 (HSP60). Hence, we hypothesised that *C. pneumoniae* infection is associated with anti-CCP antibodies.

Methods

*C. pneumoniae* IgA and anti-CCP antibody levels were determined in 26 healthy subjects in this cross-sectional study. Serum *C. pneumoniae* IgA antibody levels were assessed using an enzyme-linked immunosorbent assay. Serum anti-CCP antibody levels were assessed using fluoroenzymeimmunoassay.

Results

There was a highly significant positive correlation between the two sets of antibodies (*r_s_* = 0.621; *P* = 0.0007). Linear regression analysis showed that this correlation was not the result of age or sex.

Discussion

A biologically plausible mechanism is put forward for these results, involving HSP60 acting as an endogenous ligand for toll-like receptor 4 (TLR4) and the interaction of TLR4 with lipopolysaccharides, which occur in the outer membrane of the *C. pneumoniae* elementary body. Pronounced pro-inflammatory mediator secretion then takes place. The release of Ca^2+^ ions may then activate local peptidylarginine deiminases, leading to the formation of CCPs and thus the reported finding. Confirmation of these results may have potential clinical implications in terms of diagnosis, including pre-symptomatic diagnosis, and treatment.

## Introduction

Microbial pathogenesis has been put forward as a cause of human autoimmune disorders; in particular, it has been suggested that attention should be paid to the potential pathogenic role of the obligate intracellular bacterial species *Chlamydia pneumoniae* (*C. pneumoniae*) [1,2]. The most prevalent chronic human autoimmune disorder worldwide is rheumatoid arthritis [3], and it is noteworthy that Fujita et al. found a 30% prevalence of *C. pneumoniae* immunoglobulin M (IgM) in 27 patients in the acute phase of this illness (compared with a prevalence of 10% in controls) [1]. Furthermore, Gérard et al. reported that synovial samples from all seven patients in their cohort of subjects with either rheumatoid arthritis or undifferentiated oligoarthritis proved polymerase chain reaction (PCR)-positive for *C. pneumoniae* DNA, while samples from a cohort of two patients with osteoarthritis were PCR-negative; moreover, reverse transcription (RT)-PCR assays targeting *C. pneumoniae* rRNA operons and mRNAs demonstrated that these synovial bacteria were metabolically active [4].

The anti-cyclic citrullinated peptide (anti-CCP) antibody has emerged as an important autoantibody in the study and clinical assessment of rheumatoid arthritis [5,6]. Post-translational deamination of protein-bound arginine residues is catalysed, in a Ca2+-dependent manner, by mammalian peptidylarginine deiminases, which may cause protein unfolding [7]. Filaggrin, a filament-associated protein, is a known peptidylarginine deiminase substrate, with arginine deiminisation proceeding rapidly to greater than 95% completion [7]. Anti-CCP antibodies, particularly to (pro)filaggrin, occur in most patients with this autoimmune disorder, with a sensitivity of 67% to 83% and a specificity of 90% to 96% [5,6].

We now demonstrate that there is a link between the aforementioned findings of Gérard et al. [4] and anti-CCP autoantibodies. The RT-PCR *C. pneumoniae* gene expression studies by Gérard et al. screened four mRNA sequences [4]. Just one of these, namely hsp60, was positive in the synovial samples of all seven patients with rheumatoid arthritis/undifferentiated oligoarthritis; it was negative in the synovial samples of both osteoarthritis patients [4]. Thus, this study demonstrated the expression of heat shock protein 60 (HSP60) by *C. pneumoniae* in the synovium in this patient group. More recently, Lu et al. showed that anti-CCP antibodies promote apoptosis of mature human Saos-2 osteoblasts via cell-surface binding to citrullinated HSP60 [8].

Hence, we hypothesised that *C. pneumoniae* infection is associated with anti-CCP antibodies. Given that *C. pneumoniae* is an aetiological agent for respiratory tract infections, and given the role of IgA in mucosal secretions, we aimed to assess the correlation of *C. pneumoniae* IgA with anti-CCP antibodies [2,9-11]. The hypothesised microbial pathogenic mechanism is not dependent on pre-existing rheumatoid arthritis; it would be expected to start to take place well before the emergence of clinical symptomatology. We, therefore, examined a cohort of healthy volunteers, specifically those without any known rheumatological disorder. From a statistical viewpoint, this would also be likely to give us a greater range of values, including data points close to the origin.

## Materials and methods

Subjects

Healthy subjects aged between 18 and 80 years were recruited into the study in a British outpatient clinic. A clinical history of any rheumatological disorder was a specific exclusion criterion. The subjects were physically well. A detailed medical history was taken for each subject. Each subject underwent a physical examination by the first author.

Ethical approval for this study was received from the CPU Research Ethics Committee (approval number 001/18). The study was carried out in accordance with the Declaration of Helsinki; all subjects gave informed written consent.

Sampling and laboratory assays

Venous peripheral blood was collected from each subject directly into a BD Vacutainer SST™ II Advance tube (Becton, Dickinson, and Company, New Jersey, USA) containing a gel to enable the separation of serum. The labelling of each tube was an alphanumeric code, which maintained anonymity. Following arrival at the laboratory, serum *C. pneumoniae* IgA was assessed using an enzyme-linked immunosorbent assay (ELISA), which provided a semi-quantitative in vitro assay for human antibodies (EUROIMMUN Medizinische Labordiagnostika AG (part of PerkinElmer), Lübeck, Germany), while the fluoroenzymeimmunoassay EliA was used to quantify serum IgG antibodies directed to CCP (Thermo Fisher Scientific Inc., Waltham, MA, USA).

Statistical analysis

Two-tailed statistical testing was performed on a 64-bit x86_64-w64-mingw32/x64 platform using the programmes R 4.2.1 (The R Foundation, Vienna, Austria) and JASP 0.18.1 (The Jasp Team, Amsterdam, The Netherlands) [12,13].

## Results

Demographics summary

A total of 26 subjects were studied. A total of 25 of the subjects were female. The ages of the subjects ranged from 22 to 71 years (median age 50 years, interquartile range 14 years). All 26 subjects were White Caucasian.

Antibody values

There were no missing values. The serum IgG anti-CCP antibody levels and the serum *C. pneumoniae* IgA ELISA ratio values for each of the 26 subjects are given in Table 1.

**Table 1:** Anti-CCP antibody values and C. pneumoniae IgA ELISA ratio values for all 26 subjects CCP: cyclic citrullinated peptide; IgA: immunoglobulin A; *C. pneumoniae*: *Chlamydia pneumoniae*; ELISA: enzyme-linked immunosorbent assay

Subject number	Anti-CCP antibody/U mL^-1^	*C. pneumoniae* IgA
1	0.5	0.623
2	0.5	1.157
3	0.9	0.215
4	1.0	1.008
5	0.4	1.235
6	0.0	0.158
7	0.7	1.153
8	0.6	0.368
9	0.8	0.426
10	0.4	0.854
11	0.0	0.145
12	0.4	0.106
13	0.5	0.422
14	2.0	0.957
15	0.4	0.129
16	0.0	0.120
17	0.0	0.165
18	0.4	0.111
19	0.8	0.503
20	0.5	0.146
21	0.0	0.097
22	1.0	1.909
23	0.6	0.160
24	1.0	1.049
25	0.5	0.675
26	0.5	0.163

Correlation between *C. pneumoniae* IgA and autoantibodies

**Figure 1:**
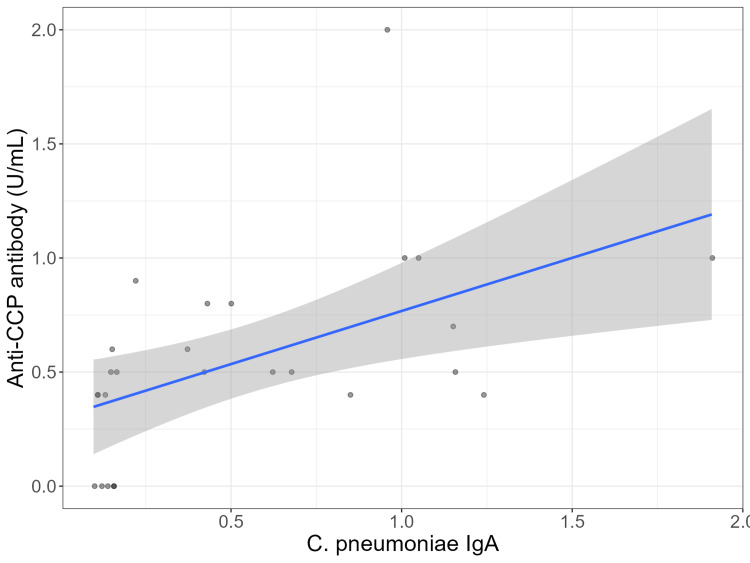
Correlation between anti-CCP antibody levels and C. pneumoniae IgA with superimposed regression line and its 95% confidence interval The continuous blue line is the best-fit regression line. It is surrounded by a grey zone which represents the 95% confidence interval of the regression line. CCP: cyclic citrullinated peptide; IgA: immunoglobulin A; C. pneumoniae: *Chlamydia pneumoniae*

The Spearman rs correlation coefficient between the two variables shown in Table 1 was calculated. It was found to be 0.621 (P = 0.0007). Figure 1 shows the correlation plot in which anti-CCP antibody levels are plotted against *C. pneumoniae* IgA values. The regression line is also shown in Figure 1 as a continuous blue line with a positive gradient. The 95% confidence interval of the regression line is shown in Figure 1 as a grey zone surrounding the regression line.

Linear regression

To adjust the results for age and sex, a linear regression analysis was carried out with anti-CCP antibody as the dependent variable, age and *C. pneumoniae* IgA as covariates, and sex as a factor. In the resulting model (F3,22 = 3.738, P = 0.026), the coefficients of both age (P = 0.643) and sex (female; P = 0.233) were not statistically significant; only the coefficient of *C. pneumoniae* IgA reached statistical significance (P = 0.004).

## Discussion

This first study of *C. pneumoniae* IgA and anti-CCP antibodies in healthy subjects shows that the two sets of antibodies are highly correlated. The linear regression analysis shows that this correlation is not the result of age or sex. Thus, these results offer support for our hypothesis.

If the correlation is the result of causation, our results do not exclude the possibility that the direction of causality may be from autoimmunity to microbial infection. In particular, it could be argued that the development of an autoimmune disorder such as rheumatoid arthritis predisposes a subject to increased susceptibility to any bacterial infection. Evidence against this is provided by the study by Fujita et al. mentioned above, in which 30% of a cohort of 27 patients in the acute phase of rheumatoid arthritis were found to be positive for *C. pneumoniae* IgM antibodies [1]. In addition to assessing patients in the acute phase of rheumatoid arthritis, they also included patients in the acute phase of several other autoimmune disorders, and in addition to assessing *C. pneumoniae*, they also assessed evidence of two other bacterial infections. Just 8% of the patients were positive for *Mycoplasma pneumoniae* IgM and only 4% for pharyngeal *Streptococcus pyogenes* antigen; these figures are much lower than those for *C. pneumoniae* IgM (30% for rheumatoid arthritis; 29% across all autoimmune disorders studied) [1]. Furthermore, our cohort of healthy subjects did not have any history of rheumatological disorder or, indeed, any autoimmune disorder.

Pharmacotherapy of autoimmune disorders with corticosteroids and disease-modifying antirheumatic drugs (DMARDs) such as tumour necrosis factor (TNF) inhibitors can increase the susceptibility to bacterial infection, including bacterial pneumonia [14-16]. It might, therefore, be argued that such medication could be a confounding variable that accounts for our finding. However, none of the healthy subjects in our cohort were taking corticosteroid treatment or DMARD treatment. Furthermore, the 27 rheumatoid arthritis patients studied by Fujita et al. (see above) were all in the acute phase of the illness, with an evaluation of their reported *C. pneumoniae* antibody titres specifically being carried out before any treatment with corticosteroids or DMARDs [1].

It has been hypothesised that other bacterial species, particularly those such as Aggregatibacter actinomycetemcomitans, which are implicated in oral infections such as periodontitis, may also play a role in the aetiology of autoimmune disorders such as rheumatoid arthritis [17,18]. Evidence put forward in support of this hypothesis is the association of periodontitis with the presence of anti-CCP antibodies and reports of the beneficial effects on rheumatoid arthritis symptomatology of treatment with antibiotics [19,20]. Interestingly, both the presence of anti-CCP antibodies and the beneficial actions of antibiotic therapy are also consistent with our hypothesis. One particular strength of our study, in comparison with those concerned with periodontal infection, is that specific *C. pneumoniae* IgA antibody levels have been assessed by us. A second strength of our study is the finding of what is essentially a dose-response type relationship between *C. pneumoniae* IgA and anti-CCP antibodies. This second strength fulfills the first criterion of causal inference, namely that of the strength of association, proposed in 1965 by the late Professor Sir Austin Bradford Hill [21]. Our earlier discussion regarding the direction of causality would lend weight to the fulfilment of Bradford Hill’s second criterion of causal inference, namely temporality; for the reasons given above, it seems probable that infection with *C. pneumoniae* precedes the development of autoantibodies [21].

A particularly important criterion of causal inference is, in Bradford Hill’s own words [21], “It will be helpful if the causation we suspect is biologically plausible.” It is to this criterion that we now turn our attention. The findings relating to HSP60 mentioned earlier provide a biologically plausible mechanism for the association between *C. pneumoniae* IgA and anti-CCP antibodies. Once a synovial joint becomes infected by *C. pneumoniae*, the synovial bacteria are viable and metabolically active, producing HSP60 [4]. In turn, HSP60 may act as an endogenous ligand for toll-like receptor 4 (TLR4) [22]. In addition, the outer membrane of the *C. pneumoniae* elementary body (the infectious form of the bacterium) is rich in lipopolysaccharides (LPS) [23]. The LPS-TLR4 axis regulates osteoclastogenesis, with pronounced pro-inflammatory mediator secretion occurring following the interaction of LPS with TLR4; such mediators include interleukin-6, TNF-α, and interleukin-1 [24-26]. It seems reasonable to postulate that the release of Ca2+ ions subsequent to inflammatory osteolysis can activate local peptidylarginine deiminases, which are Ca2+-dependent [5,27,28]. In turn, this would lead to the formation of CCPs [5,28], hence the association between *C. pneumoniae* IgA and anti-CCP antibodies.

Limitations

This novel study is cross-sectional in nature. It would be useful to assess the differential development, in relation to baseline antibody levels, of repeated antibody assays and the symptomatology of autoimmune disorders over several years. In order to do this, the present study could be extended into a longitudinal study.

## Conclusions

This is the first study of *C. pneumoniae* IgA and anti-CCP antibodies in healthy subjects. The level of the *C. pneumoniae* antibodies was highly correlated with the level of the anti-CCP autoantibodies. The direction of causality is very likely to be from the former to the latter. *C. pneumoniae* infection appears to lead to the formation of autoantibodies. A biologically plausible mechanism for this process has been put forward in this paper. Confirmation of the role of *C. pneumoniae* in the aetiology of autoimmune disorders would be of value in diagnosing autoimmune disorders. It would also be of value in carrying out pre-symptomatic diagnostic testing, perhaps leading to the implementation of screening programmes. Finally, it may transpire that successful treatment of *C. pneumoniae* infections may be beneficial for the treatment of associated autoimmune disorders.
